# Full-Length Transcriptome Analysis Reveals Candidate Genes Involved in Terpenoid Biosynthesis in *Artemisia argyi*

**DOI:** 10.3389/fgene.2021.659962

**Published:** 2021-06-22

**Authors:** Yupeng Cui, Xinqiang Gao, Jianshe Wang, Zengzhen Shang, Zhibin Zhang, Zhenxing Zhou, Kunpeng Zhang

**Affiliations:** ^1^College of Biology and Food Engineering, Anyang Institute of Technology, Anyang, China; ^2^State Key Laboratory of Cotton Biology, Institute of Cotton Research of Chinese Academy of Agricultural Sciences, Anyang, China

**Keywords:** *Artemisia argyi*, transcriptome, terpenoid biosynthesis, SMRT sequencing, gene expression patterns

## Abstract

*Artemisia argyi* is an important medicinal plant widely utilized for moxibustion heat therapy in China. The terpenoid biosynthesis process in *A. argyi* is speculated to play a key role in conferring its medicinal value. However, the molecular mechanism underlying terpenoid biosynthesis remains unclear, in part because the reference genome of *A. argyi* is unavailable. Moreover, the full-length transcriptome of *A. argyi* has not yet been sequenced. Therefore, in this study, *de novo* transcriptome sequencing of *A. argyi*'s root, stem, and leaf tissues was performed to obtain those candidate genes related to terpenoid biosynthesis, by combining the PacBio single-molecule real-time (SMRT) and Illumina sequencing NGS platforms. And more than 55.4 Gb of sequencing data and 108,846 full-length reads (non-chimeric) were generated by the Illumina and PacBio platform, respectively. Then, 53,043 consensus isoforms were clustered and used to represent 36,820 non-redundant transcripts, of which 34,839 (94.62%) were annotated in public databases. In the comparison sets of leaves vs roots, and leaves vs stems, 13,850 (7,566 up-regulated, 6,284 down-regulated) and 9,502 (5,284 up-regulated, 4,218 down-regulated) differentially expressed transcripts (DETs) were obtained, respectively. Specifically, the expression profile and KEGG functional enrichment analysis of these DETs indicated that they were significantly enriched in the biosynthesis of amino acids, carotenoids, diterpenoids and flavonoids, as well as the metabolism processes of glycine, serine and threonine. Moreover, multiple genes encoding significant enzymes or transcription factors related to diterpenoid biosynthesis were highly expressed in the *A. argyi* leaves. Additionally, several transcription factor families, such as RLK-Pelle_LRR-L-1 and RLK-Pelle_DLSV, were also identified. In conclusion, this study offers a valuable resource for transcriptome information, and provides a functional genomic foundation for further research on molecular mechanisms underlying the medicinal use of *A. argyi* leaves.

## Introduction

The genus *Artemisia argyi* Lev. et Vant. (*A. argyi*) consists of ~350–500 species and is the largest in the family *Asteraceae* (Bora and Sharma, 2011). *Artemisia* species are widely distributed in the temperate zones of the Northern Hemisphere, such as North America, Europe and Asia (Riggins and Seigler, 2012), with a few species in the Southern Hemisphere (Weston et al., 2005). *A. argyi* is an important medicinal plant widely utilized for moxibustion heat therapy in China. Many of them are used as forage for animals, food, ornamental plants, or soil stabilizers in different geographical regions (Abad et al., 2012; Alok et al., 2016); in China, 17 species of *Artemisia* could be used for mugwort (Du et al., 2021). Several of these plant species have a broad range of therapeutic functions linked to their high content of essential oils and terpenoids; these include anti-bacterial, anti-malarial, cough relief, anti-cancer, anti-diabetes, and anti-diarrhea properties, as well as enhancing immunity (Meng et al., 2018; Jiang et al., 2019; Qadir et al., 2019). For example, artemisinin is a sesquiterpene lactone drugs with anti-malaria properties and it is widely used against malaria (Duffy and Mutabingwa, 2006; Gaur et al., 2014).

*A. argyi* has a long history of being used as medicine, and the whole plant of whom has a variety of bioactive compounds with amazing pharmacological effects. For example, the chemical compositions of *A. aegyi* leaves are mainly volatile oil, flavonoids, organic acids, eudesmane, terpenoids, etc. (Du et al., 2021). As the saying goes, there are 3 years of *A. argyi* home, the doctor does not have to come, which is the greatest affirmation for its medicinal value. “Chinese Mugwort” (*A. argyi*) is the earliest and most widely used species in China. And Chinese mugwort produced in the hometown of the world-famous medical scientist Li Shizhen in Hubei is the preferred variety with a long history of its medicinal and aromatic characteristics, also known as “Qi Ai” (Bora and Sharma, 2011).

Many secondary metabolites with terpenoid chemical properties play a vital role in human health and nutrition, and terpenoids also form the chemical constituents of volatile oils, resins, and waxes (Danova et al., 2018). It is reported that terpenoid biosynthesis occurs in the cytoplasm and plastids, and all terpenoids are formed by the condensation of isopentenyl diphosphate (IDP) and its allylic isomer dimethylallyl diphosphate (DMADP) (Carretero-Paulet et al., 2002; Aharoni et al., 2006). Both IDP and DMADP are synthesized in plastids by the 2-methylerythritol 4-phosphate pathway (MEP) via deoxy-D-xylulose 5-phosphate, but IDP is also synthesized in the cytoplasm by the mevalonate pathway (Bick and Lange, 2003). Mugwort volatile oil (MVO), a traditional Chinese herbal medicine, can have anti-bacterial and antiviral effects and may help ameliorate stomachaches and bleeding (Kim et al., 2006). It was recently reported that MVO has a noticeable killing effect on human demodicid mites (Du et al., 2021), and its secondary metabolites are known to be responsible for a variety of important pharmacological properties (e.g., larvicidal, insecticidal; Wang et al., 2006). Moreover, both traditional and modern pharmaceutical systems imply its uses in the treatment of gynecological problems (Deoray and Page, 2018).

Given the demand for important medicinal metabolites, it is necessary to study the metabolite pathways and the related genes involved in them, for which the most effective approach is analyzing transcriptome data. However, biological molecular studies of Chinese mugwort lag far behind those of its chemistry and pharmacology (Jiang et al., 2019; Gao et al., 2020). Transcriptome sequencing is currently the best technique available to analyze functional genes and gene expression patterns. In this respect, however, only small portions of *Artemisia*'s chloroplast genome and transcriptome have been sequenced (Danova et al., 2012, 2018; Srivastava and Sangwan, 2012; Kim et al., 2020; Ma et al., 2020; Shahzadi et al., 2020), and only for *A. annua* has a draft nuclear genome sequence been fully reported (Shen et al., 2018). The advent of omics sequencing technology has promoted the rapid development of chloroplast genomics and genetics of medicinal plants, so for species whose genomic information is unavailable, high-throughput RNA sequencing and data analysis are powerful tools for mining genes with specific biological functions.

Here we obtained candidate transcripts and transcription factors (TFs) involved in the biosynthesis process of terpenoids in *A. argyi*, by combining RNA-Seq and SMRT sequencing analysis. The results offer a valuable resource for future elucidating the molecular mechanisms in the medicinal use of *A. argyi* leaves.

## Materials and Methods

### Plant Materials and RNA Extraction

The *A. argyi* plants (Chinese mugwort) were taken from tissue cultures and brought into a greenhouse, where they were placed in a controlled environment growth chamber for 1 month of cultivation under a 12-h/12-h photoperiod on a 25°C/25°C days/night cycle. Then, we selected three *A. argyi* plants similar in size and collected their root, stem, and leaf parts; these tissue samples were immediately frozen in liquid nitrogen and stored at −80°C until the RNA extraction. A total of nine RNA samples of root, stem and leaf tissues (each with three biological replicates) were isolated using Trizol (Invitrogen, USA). The total RNA samples were treated with Dnase I to remove any DNA contaminants in them. The quantity and quality of the extracted RNA were determined using Nanodrop and agarose gel, and further checked with an Agilent 2100 Bioanalyzer according to the manufacturer's instructions.

### Library Construction and Transcriptome Sequencing

After extracting the total RNA from the root, stem, and leaf parts of *A. argyi* (with three biological repetitions), nine libraries were constructed for Illumina transcriptome library construction and sequencing. Random hexamer primers and Superscript III reverse transcriptase were used to synthesize the first-strand cDNA. The RNA templates were later removed, and then RNase H (Invitrogen) and DNA polymerase I were used to synthesize the second-strand cDNA. The concentration and quality of these short double cDNA fragments was respectively measured by Qubit 2.0 and Agilent 2100 Bioanalyzer. Finally, the nine libraries were sequenced from both the 5′ and 3′ ends by Biomarker Technologies (Beijing, China), using the Illumina NovaSeq 6000 platform with paired-end (PE) approach and read length of 125 nt. The unigenes assembled from the Illumina short reads by Trinity (Grabherr et al., 2011). The flowchart of bioinformatics analysis for full-length transcriptome was shown in Supplementary Figure 1.

For the PacBio SMRT full-length transcript library construction and sequencing, equal quantities of total RNA from the nine individual samples described above were pooled following the manufacturer's instructions. Then library was sequenced on the PacBio platform (Pacific Biosciences, Menlo Park, CA, USA), using four SMRT cells. The integrity of de-redundant transcriptome was evaluated using BUSCO (Simão et al., 2015). TransDecoder (https://github.com/TransDecoder/TransDecoder/releases) was used to identify candidate coding regions within transcript sequences.

### Detection of Alternative Splicing Events, lncRNAs, and Transcripts Factors

Raw reads were processed into circular consensus (CCS) read according to the adaptor by SMRT analysis software package (Gordon et al., 2015). Next, full-length non-chimeric (FLNC) transcripts were determined by searching for the polyA tail signal and the 5′ and 3′ cDNA primers in CCSs. Consensus isoforms were obtained through grouping the similar full-length sequences, or the full-length sequences from the same transcript, into a cluster. All the FL consensus sequences were first corrected using both Quiver and the Proofreads tool based on the Illumina short reads (Hackl et al., 2014), to obtain high-quality sequences (i.e., identity > 0.99) for subsequent analyses. Finally, the longest isoform of each cluster was considered as the transcript after removing redundancies with CD-HIT software (Li and Godzik, 2006).

The alternative splicing (AS) events, including alternative exon ends (5′, or 3′, or both) (AE), intron retention (IR), skipped exon (SKIP), transcript start site (TSS), and transcript terminal sit (TTS) were obtained as previously described (Zhang et al., 2018). Briefly, these AS events were identified using the Cuffdiff tool, based on both the SMRT and Illumina transcriptome data. Transcripts showing insufficient read coverage (i.e., read numbers < 30) at junction sites and putative AS events, which would result in very short proteins in the alternate ORF, were excluded (Schliebner et al., 2014). The identification of lncRNAs was carried out using SMRT RNA-Seq data, as previously described (Ou et al., 2017). Briefly, all the transcripts with strand information and transcripts don't overlapped with known genes were retained. The transcripts of longer than 200 nucleotides with FPKM > 0.5 in multiple-exons or 2 in single-exion in at least one sample were retained. The CPC (Coding Potential Calculator) and CNCI (Coding Non-Coding Index) program were used to predict the coding potential of the remaining transcripts. All the transcripts with CPC scores > 0 or CNCI > 0 were removed. Finally, the retaining transcripts were candidate lncRNAs. TFs were predicted using the PlantTFDB (http://planttfdb_v4.cbi.pku.edu.cn/) (Jin et al., 2017).

### Quantification of the Expression Profile and Identification of Differentially Expressed Transcripts (DETs)

Clean reads were obtained after removing any low-quality reads and those reads containing adapters and poly-N with Trimmomatic 0.4 (Bolger et al., 2014). Gene expression levels were identified by calculating the total numbers of the fragments mapping to each transcript and then converting these fragment counts to fragments per kilobase of transcript per million mapped reads (FPKM) for their normalization. Differential expression analysis was performed using the DESeq2 package (Anders and Huber, 2010). The resulting *P*-values were adjusted using the Benjamini and Hochberg approach (Benjamini and Hochberg, 1995) for controlling the false discovery rate (FDR). Genes with FDR < 0.01 and fold-change ≥ 2 or ≤ −2 found by DESeq2 were designated as those differentially expressed.

### Functional Annotation and Classification of Transcripts

Non-redundant transcripts were annotated and searched against eight databases: Nr (NCBI non-redundant protein sequences), GO (Gene Ontology) (Ashburner et al., 2000), Swiss-Prot (Apweiler et al., 2004), Clusters of Orthologous Groups of proteins (COG) (Tatusov et al., 2000), evolutionary genealogy of genes: Non-supervised Orthologous Groups (eggNOG) (Huerta-Cepas et al., 2016), euKaryotic Ortholog Groups (KOG) (Koonin et al., 2004), KEGG (Kyoto Encyclopedia of Genes and Genomes) (Kanehisa et al., 2004), and Pfam (El-Gebali et al., 2018). In all these searches, an E-value of 10^−5^ was the cutoff.

### GO and KEGG Pathway Enrichment Analysis of DETs

The GO and KEGG pathway enrichment analysis of DETs were, respectively, carried out by the gProfiler package and KOBAS 3.0 (Xie et al., 2011). For this, the Benjamini–Hochberg method was also used to conduct the multiple testing in a corrected manner (Benjamini and Hochberg, 1995) by controlling the FDR (5%). Additionally, the small term cutoff value was set to five.

### Quantitative RT-PCR (qRT-PCR) Validation

To confirm RNA-Seq results, the expression of 12 randomly-selected genes were analyzed by qRT-PCR, using the same RNA samples as those used for transcriptome sequencing. Total RNA of all samples was isolated with Trizol (Invitrogen), and from each ~0.5 μg of RNA was used for the synthesis of first-strand cDNAs with the PrimerScript 1st Strand cDNA synthesis kit (TaKaRa, Dalian, China), by following the manufacturer's protocol. The specific primers used are listed in Supplementary Table 1; *AaActin* gene served as an internal reference to normalize all the data, after which gene expression levels were calculated using the 2^−ΔΔCt^ method (Livak and Schmittgen, 2001). Three biological replicates and three technical replicates were conducted for each selected-gene. Based on the internal reference of *AaActin* gene, we added two other internal parameters *RPII* (F01_transcript_15723) and *EF1*α (F01_transcript_29404) for primer specific amplification through transcriptome data in this study, the results showed that all the three reference genes amplified a single band, and the melting curve had only one obvious peak. The stability of the three reference genes was evaluated by geNorm software, and the M value of the three genes were less than 1.5, and the M value of *Actin* was the smallest, followed by *EF1*α and *RPII*. The amplification efficiency of the primers used in this study was analyzed by standard curve method, and the amplification efficiency of the primers is available in Supplementary Table 1.

## Results

### Transcriptomic *de novo* Assembly From SMRT Sequencing and Short Reads in *A. argyi*

To comprehensively identify and differentiate the gene expression profile in leaves from other plant parts of *A. argyi*, the transcriptomes of root, stem, and leaf tissues of *A. argyi* were generated simultaneously by full-length transcript analysis with PacBio SMRT sequencing and *de novo* transcript assemblies with NGS Illumina paired-end RNA-Seq reads. The mRNAs of these root, stem and leaf samples (each having three biological replicates) were first subjected to the Illumina NovaSeq 6000 platform for PE 150-bp sequencing; this yielded a total of 55.4 Gb clean reads, as each sample produced more than 6 Gb after the quality filtering process. The average GC percentage and Q30 of all libraries was 42.78 and 94.15, respectively (Supplementary Table 2). The integrity of transcriptome was shown in Supplementary Figure 2. Subsequently, the pooled full-length cDNAs from all samples were normalized and used for the PacBio SMRT sequencing. Total 139,309 Reads of Insert (ROI) were successfully extracted in the SMRT sequencing with 45 passes and mean length of 2,441 bp, including 108,846 full-length ROIs (78.13%). Moreover, all ROIs were clustered and 53,043 consensus isoforms were determined, including 52,371 high-quality and 613 low-quality transcripts with differential patterns of length distribution (Table 1). To further improve their quality, the CD-HIT (c = 0.90) was used to remove redundant sequences in all high-quality transcripts and corrected low-quality transcripts, based on the Illumina RNA-Seq reads, whose sequencing depth and accuracy are higher than that of SMRT sequencing. Finally, 36,820 non-redundant transcripts ranging in length from 320 to 42 890 bp were generated for the following analyses (Supplementary Figure 3).

**Table 1:** Summary of PacBio single-molecule real-time (SMRT) sequencing.

**cDNA size**	**Number of unpolished consensus isoforms**	**Mean unpolished consensus isoform read length**	**Number of polished high-quality isoforms**	**Number of polished low-quality isoforms**	**Percent of polished high-quality isoforms (%)**
1–6 K	53,043	2,461	52,371	613	98.84
All	53,043	2,461	52,371	613	98.84

### Functional Annotation of Identified Transcripts

**Figure 1:**
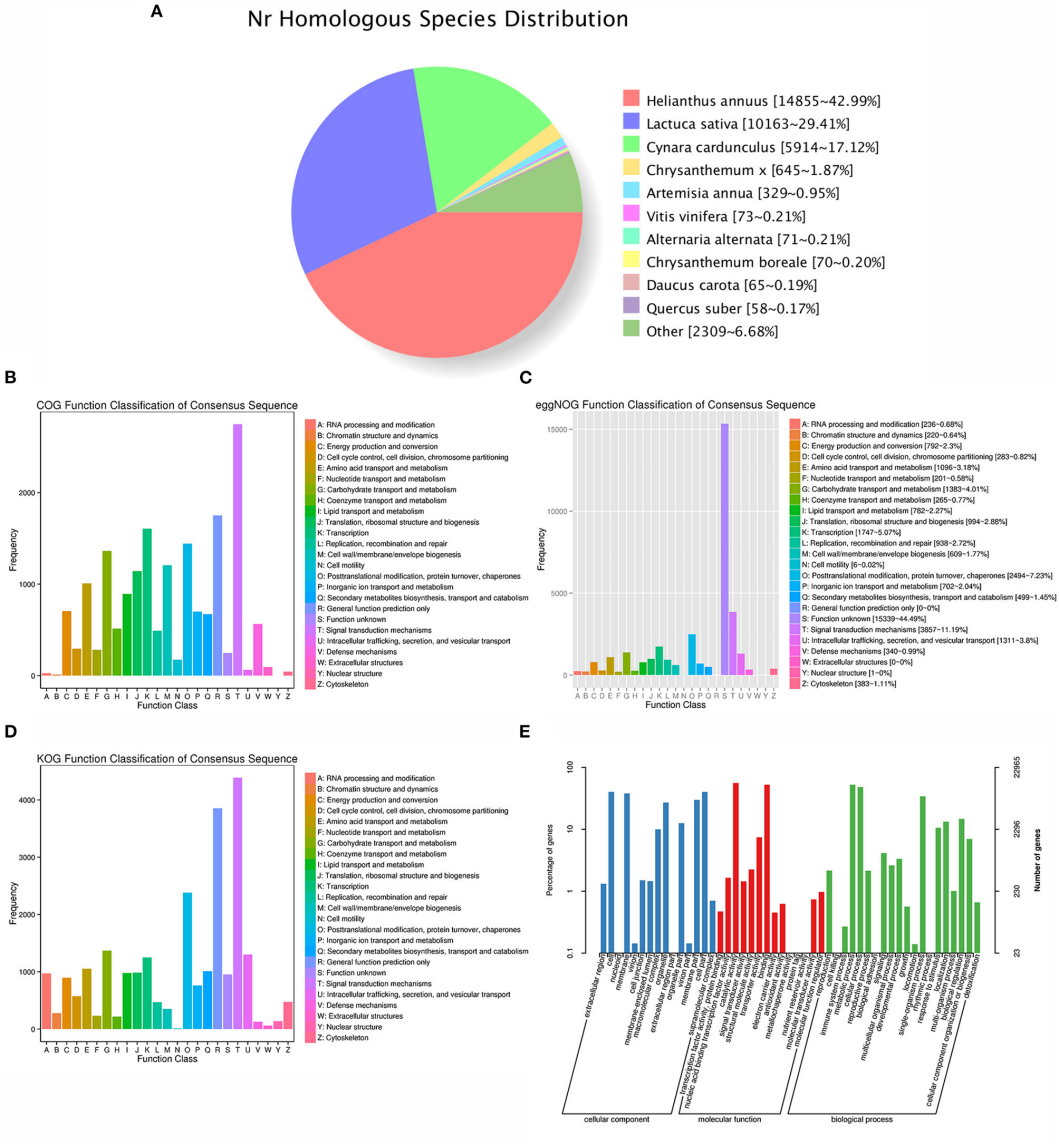
Functional annotation of *A. argyi* transcripts. **(A)** All transcripts were assigned to database Nr. **(B)** All transcripts were assigned to database COG and classified into 24 functional categories. **(C)** All transcripts were assigned to eggNOG categories and classified into 23 functional terms. **(D)** All transcripts were assigned to database KOG and classified into 25 functional categories. **(E)** All transcripts were assigned to database GO.

For the functional annotation, the different domain/family of distinct gene sequences were first searched against public databases by BLAST. Among the 36,820 identified transcripts, 16,993 (46.15%), 22,965 (62.37%), 15,688 (42.61%), 22,404 (60.85%), 29,721 (80.72%), 27,548 (74.82%), 33,807 (91.82%), and 34,599 (93.97%) acquired significant hits in the public databases of COG, GO, KEGG, KOG, Pfam, SwissProt, eggNOG, and Nr, respectively (Figure 1; Supplementary Table 3). Moreover, a total of 34,839 (94.62%) transcripts successfully annotated (Supplementary Table 3). In particular, the GO terms indicated that the functions of these transcripts and their gene products were further divided into three categories, cellular locations, biological processes and molecular functions, which comprising 49 functional enrichment categories (Figure 1E; Supplementary Table 4). In the biological process class, metabolic process (GO:0008152), cellular process (GO:0009987), and single-organism process (GO:0044699) ranked in the top three, indicating the importance of them for *A. argyi* tissues.

### Identification and Functional Annotation of DETs

**Figure 2:**
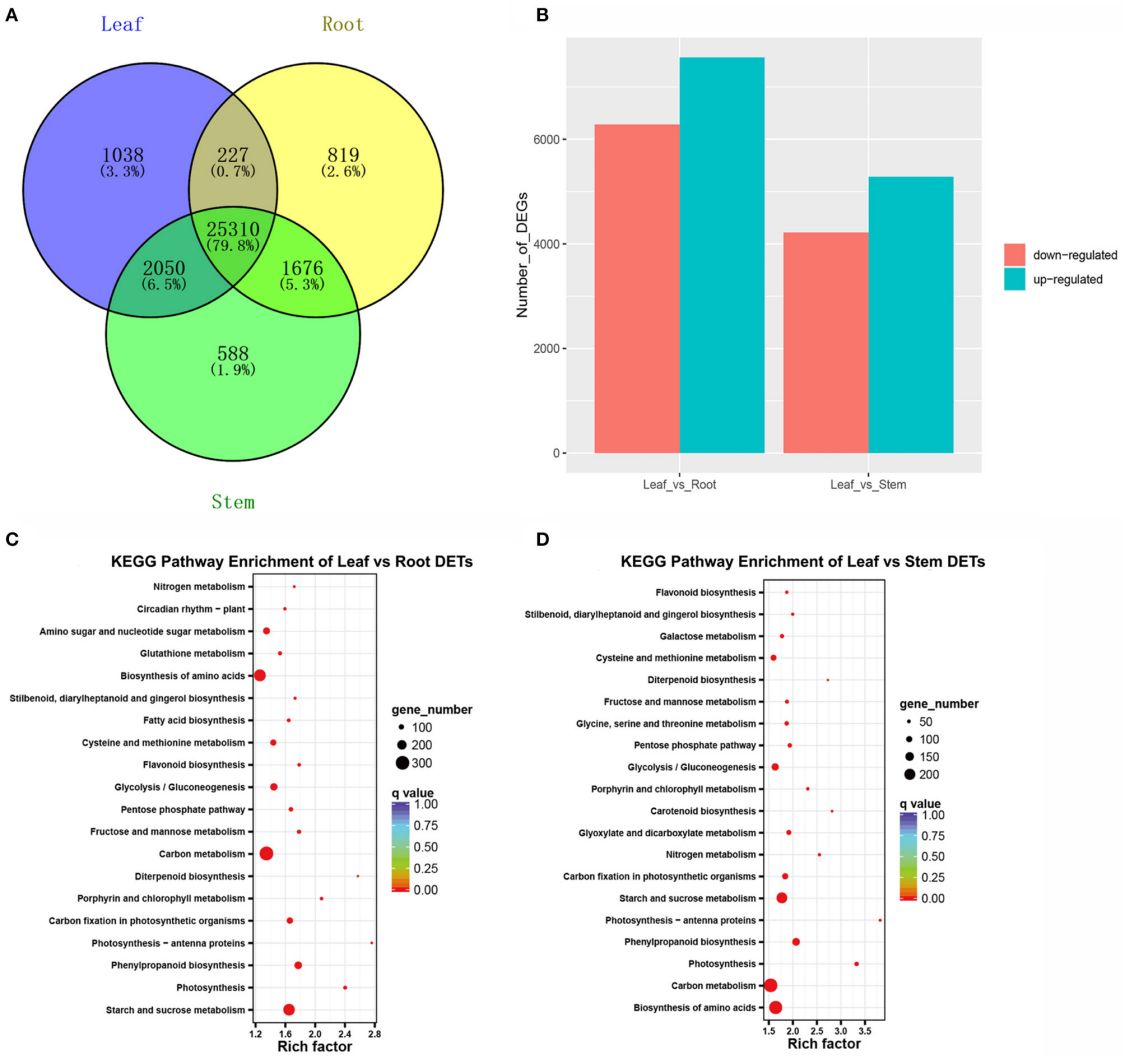
Overview of transcripts expressed in the three *A. argyi* tissues. **(A)** Venn diagram of expressed transcripts (FPKM > 1) number distributions in the three tissues. **(B)** Statistic of DETs in *A. argyi* root, stem, and leaves tissues. The numbers of up-regulated and down-regulated transcripts between the two indicated samples are summarized. DETs with lower expression levels in leaves than in roots or stems were defined as “up-regulated”, while those with higher expression levels in leaves were defined as “down-regulated”. **(C)** KEGG pathway enrichment analysis of DETs between leaf and root. **(D)** KEGG pathway enrichment analysis of DETs between leaf and stem.

To determine the gene expression profiles of *A. argyi* leaf, root, and stem tissues, their FPKM values were calculated and used to estimate the expression levels of the non-redundant transcripts. Using a cut-off of FPKM > 1, a total of 31,708 expressed transcripts were identified in all the three tissues, and 28,032, 29,624, and 28,625 expressed in the root, stem, and leaf samples, respectively (Supplementary Table 5). Notably, 1,038 transcripts were specifically expressed in the leaves (Supplementary Table 6). Venn diagrams further illustrated the number of expressed transcripts shared between two tissues or specificity expressed in each tissue (Figure 2A). In addition, the comparison of gene expression between leaf and the other tissues were also performed. The comparison of the leaves and roots found that 13,850 DETs were identified, of which 7,566 were up-regulated and 6,284 were down-regulated in the roots (Figure 2B; Supplementary Table 7). The comparison of leaves and stems revealed 9,502 DETs, of which 5,284 were up-regulated and 4,218 were down-regulated in the stems (Figure 2B; Supplementary Table 8). All these DETs were submitted to KEGG enrichment analysis for clarifying their potential biological functions. These results showed that the DETs were significantly enriched in the top 20 KEGG metabolic pathways, including biosynthesis of amino acids, carbon fixation in photosynthetic organisms, carotenoid biosynthesis, cysteine and methionine metabolism, diterpenoid biosynthesis, flavonoid biosynthesis, fructose and mannose metabolism, galactose metabolism, and the metabolism of glycine, serine, and threonine (Figures 2C,D).

### Candidate Genes and Transcription Factors Participating in Terpenoid Biosynthesis

**Figure 3:**
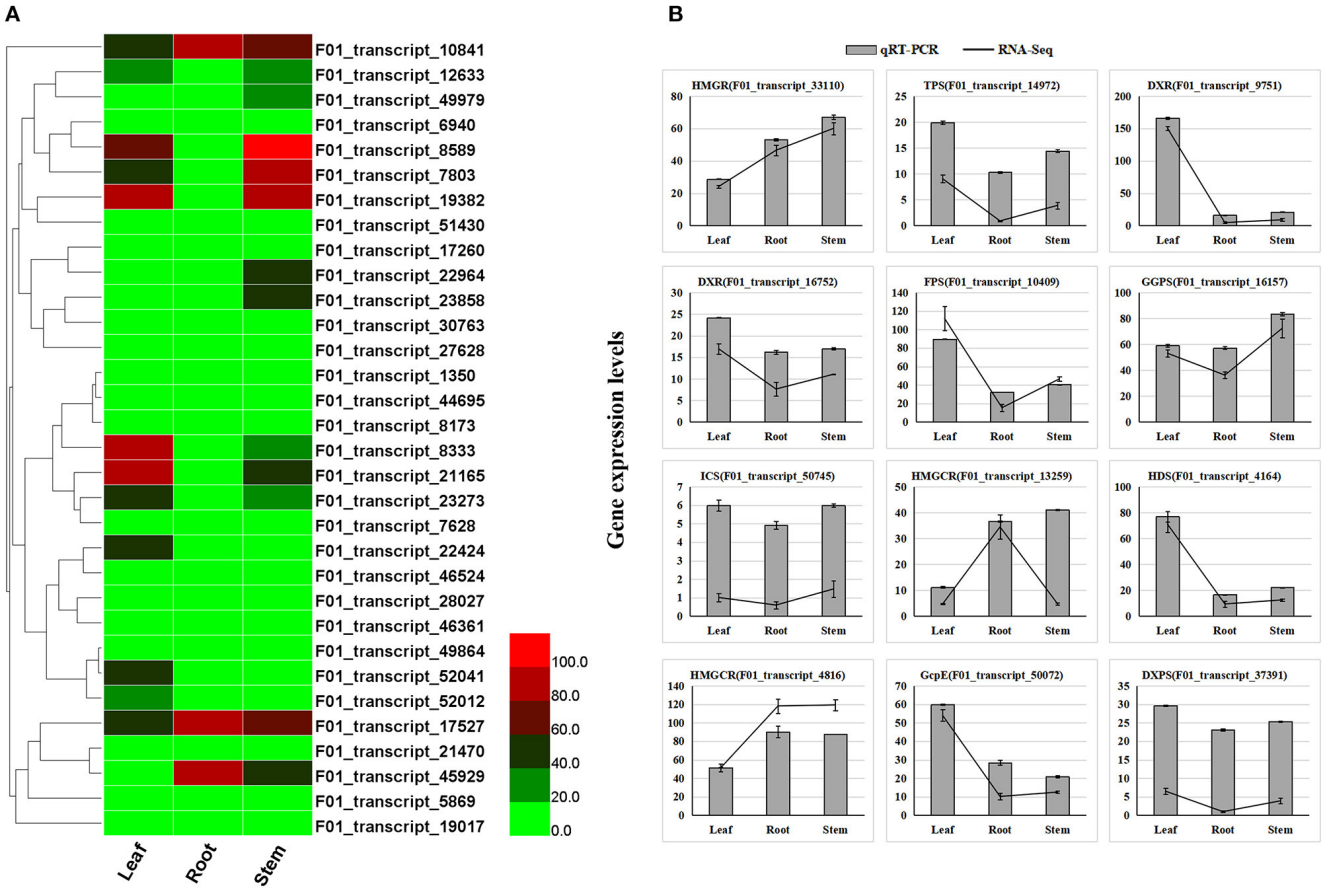
Expression profiles of terpenoid biosynthesis related transcripts in different tissues of *A. argyi*. **(A)** The expression profile of 32 candidate transcripts involved in terpenoid biosynthesis in *A. argyi*. **(B)** A comparison of the expression profiles of the 12 selected candidate transcripts as determined by RNA-Sequencing and qRT-PCR.

Leaves of *A. argyi*, which contain large amounts of volatile oils, have been extensively used for moxibustion. Additionally, moxa floss, which can be used for moxibustion as a TCM (traditional Chinese medicine) therapy to cure dysmenorrhea, diarrhea, and fatigue, can be derived from dried *A. argyi* leaves. Supplementary Table 3 shows that 15,688 (42.61%) transcripts were annotated in the KEGG database, of which 187 were involved in terpenoid biosynthesis pathways (Supplementary Table 9). Accordingly, we next wanted to identify some DETs involved in terpenoid biosynthesis pathways. The KEGG pathway enrichment analysis of all DETs found that they were mainly enriched in the biosynthesis of amino acids, carbon fixation in photosynthetic organisms, carotenoid biosynthesis, cysteine and methionine metabolism, diterpenoid biosynthesis, flavonoid biosynthesis, fructose and mannose metabolism, and galactose metabolism (Figures 2C,D). Importantly, 32 candidate genes involved in diterpenoid biosynthesis were distinguishable based on the results of this KEGG pathway enrichment analysis, which were considered as important candidate genes related to terpenoid biosynthesis in *A. argyi* (Supplementary Table 10). These 32 candidate genes were identified to encode several key enzymes that participate in the MEP and MVP pathways upstream of terpenoid biosynthesis, namely 2-C-methyl-D-erythritol 4-phosphate cytidylyltransferase, 4-hydroxy-3-methylbut-2-enyl diphosphate synthase, copalyl pyrophosphate synthase 1, 2-C-methyl-D-erythritol 2,4-cyclodiphosphate synthase, cytochrome P450 mono-oxygenase, isopenicillin N synthase, nt-kaur-16-ene synthase, chloroplastic-like isoform, 4-diphosphocytidyl-2-C-methyl-D-erythritol kinase, gibberellin 2-beta-dioxygenase, gibberellin 2-beta-dioxygenase, ent-kaurene synthase, putative oxoglutarate/iron-dependent dioxygenase, and ent-copalyl diphosphate synthase. Moreover, the transcriptome expression analysis of these candidate transcripts showed that F01_transcript_23273, F01_transcript_22424, F01_transcript_52041, F01_transcript_21165, F01_transcript_8333, F01_transcript_19382, F01_transcript_7803, and F01_transcript_8589 were all highly expressed in the leaf tissue of *A. argyi* when compared to the other two tissues (Figure 3A). This indicated they could play pivotal roles in the synthesis of terpenoids in *A. argyi* leaves. To validate the quality of our RNA-Seq results, 12 terpenoid biosynthesis related candidate genes were randomly selected for their expression analysis by qRT-PCR method. The relative expression levels of these selected genes were strongly consistent with their corresponding FPKM values (Figure 3B), thus confirming the validity of the RNA-Seq data. These candidate genes were almost highest expressed in the leaf tissues, and lowest expressed in the root tissues during the synthesis of terpenoids in *A. argyi*.

**Figure 4:**
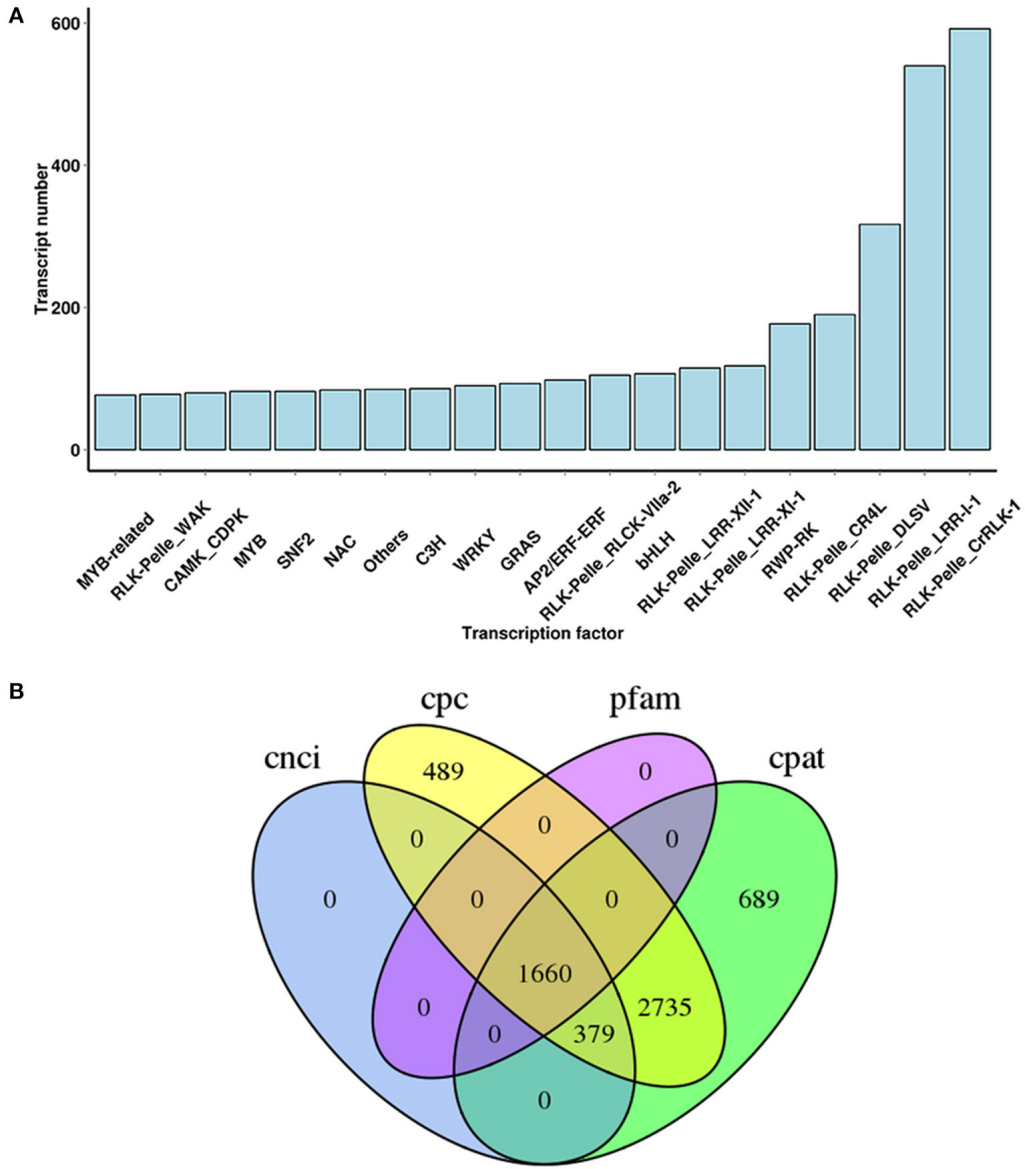
TF family classification **(A)** and predicted lncRNA **(B)** in *A. argyi*.

TFs participate in a wide variety of biological processes and play major roles in regulating gene expression at the transcriptional level. To further investigate the TFs possibly involved in terpenoid biosynthesis process in *A. argyi*, a sequence comparative analysis of all uniquely assembled transcripts with all public TFs downloaded from PlantTFDB was conducted. From this, a total of 5,604 transcripts encoding TFs were uncovered and classified into 20 different TF families (Figure 4A). Among them, the most abundant were the RLK-Pelle_CrRLK-1, RLK-Pelle_LRR-l-1, and RLK-Pelle_DLSV families of TFs.

### Identification of AS Events and lncRNAs

Pre-mRNA generated by gene transcription can undergo a variety of splicing methods. Different exons may be selected to produce different mature mRNAs, which are translated into different proteins and contributed to the diversity of biological traits. This way of processing mRNA post-transcription is called alternative splicing (AS). Candidate genes for AS events were identified by a significant read coverage within intron sequences when compared with the read coverage of the surrounding exons. In this study, omitting the protein-coding transcripts, a total of 1,660 candidate lncRNAs were also predicted by integrating four computational approaches: CPC, CNCI, CPAT, and pfam database. Some of them were predicted to be cis- and trans- regulators of the DETs, involved in metabolic processes and plant responses to stimuli (Figure 4B, Supplementary Table 11). Furthermore, a total of 1,518 alternative splicing events were detected from the non-redundant transcripts, when using the Iso-Seq data directly to run the all-vs.-all BLAST with high identity settings, namely alternative exon ends (AE), skipped exon (SKIP), intron retention (IR), transcript terminal sit (TTS), and transcript start site (TSS) (Supplementary Table 12). These AS events were distributed across 2,225 genes, of which 1,794 (80.63%) were expressed in the root, stem, or leaf parts of *A. argyi*.

## Discussion

*A. argyi* is a perennial herb, belonging to the family Asteraceae and the genus Artemisia, and is widely cultivated in China (Bao et al., 2013). The leaves of *A. argyi* have been used historically and extensively for a form of TCM known as moxfibustion (Ge et al., 2016). Previous studies of *A. argyi* leaves documented many volatile oils in them, which have antifungal, antihistamine (Edris, 2007), and antiviral effects—as well as being able to eliminate coughing and phlegm and to relieve asthma (Huang et al., 2012; Hu et al., 2013). Additionally, when ground, dried *A. argyi* leaves are the original material for moxa floss; this is often used for moxibustion as a TCM therapy to cure diarrhea (Bao et al., 2016), dysmenorrhea (Jeong et al., 2007), and fatigue (Shu et al., 2016). Moreover, high concentrations of the terpenoids could raise the economic value of *A. argyi* leaves. Although Liu et al. (2018) carried out a study about transcriptome sequencing of *A. argyi* root, stem and leaf based on just Illumina sequencing platform, the candidate genes involved in the terpenoid biosynthesis process in *A. argyi* leaves are still unclearly known.

To clarify the possible molecular regulatory mechanism of terpenoid biosynthesis in *A. argyi*, tissues corresponding to three different plant parts—roots, stems and leaves—were sampled (with three biological replicates) for their respective library construction and, for the first time, subjected to RNA-Seq and SMRT sequencing. Overall, ~55.4 Gb of clean reads were obtained from the three tissues, and 36,820 non-redundant transcripts were assembled, among which nearly all, that is 34,839 (94.62%), were annotated based on leading public databases. These annotated transcripts could provide information for the analysis and identification of crucial genes in *A. argyi*, including those encoding ribosome-inactivating proteins and lectins, which are potential anticancer drugs (Shin et al., 2017; Sindhura et al., 2017). Furthermore, functional GO annotations in terms of the cellular components, biological processes, and molecular function categories were also conducted for these non-redundant transcripts. That so many different GO terms were annotated highlights the genes' diversity likely present in the root, stem and leaf transcriptomes of *A. argyi* plants. Moreover, we have compared our sequence data to a preivous paper that also did a transcriptome analysis in the same tissues of the same species (Liu et al., 2018*)*. By re-analyzing Liu et al. (2018) data using the same approach in this study, we found that there are 99,807 and 36,820 transcripts identified using Liu et al. (2018) data and our data, respectively. The expressed transcripts are 88,592 and 36,820 in Liu et al. (2018) and our study, respectively. Candidate unigenes involved in the terpenoid biosynthesis pathway are 114 and 187 in Liu et al. (2018) and our study, respectively. The expression trends of key candidate terpenoid-related genes were basically consistent between these two studies.

**Figure 5:**
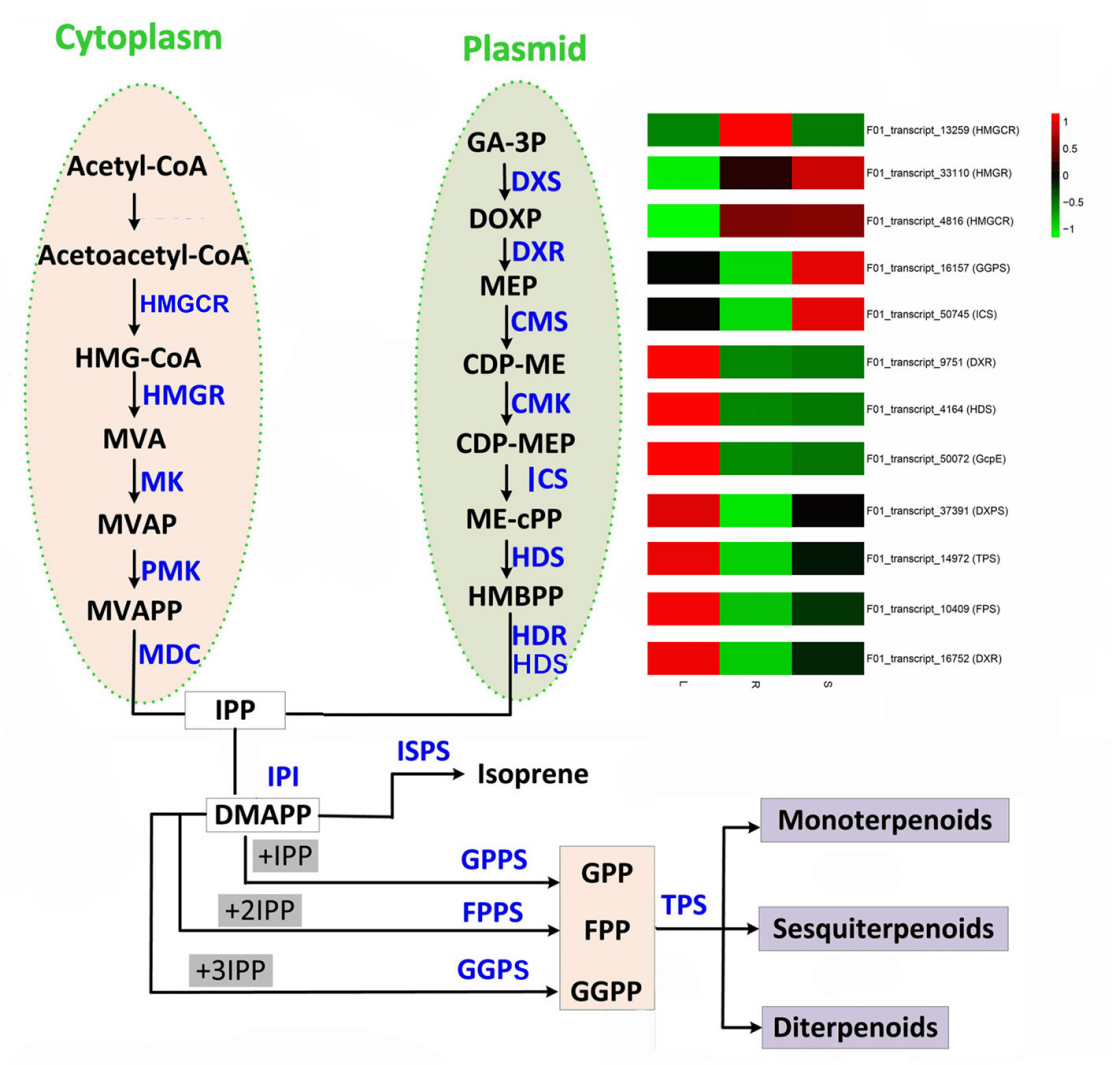
Terpenoid biosynthesis pathway in *A. argyi*.

The key terpenoid precursor IPP is synthesized by mevalonate (MVA) pathway and methyl erythritol phosphate (MEP) pathway, two independent pathways (Figure 5). *A. argyi* possess both the MVA and MEP pathways often in the same cell type. In the MVA pathway, three acetyl CoA molecules are condensed to give 3-hydroxy-3-methylglutaryl CoA (HMGCoA) by acetoacetyl CoA synthase and HMGCoA synthase. Overexpression of HMGCoA reductase without its feedback-sensing N-terminal domain is known to dramatically increase the MVA metabolic flux in engineered yeast. The MEP pathway is entirely independent of the MVA pathway. The first intermediate, 1-deoxy-D-xylulose-5-phosphate (DXP), is channeled to both IPP and pyridoxal phosphate (vitamin B6) synthesis. However, the second intermediate, MEP, is a committed molecule only for IPP biosynthesis (Figure 5). By mapping these DETs to the KEGG database, our results show that there are 187 candidate transcripts involved in terpenoid biosynthesis. In particular, some candidate genes of them were identified to encode several key enzymes that participate in the MEP and MVP pathways of terpenoid biosynthesis, such as MEP, HMGS/HMGR, FPS, CMS, DXS, TPS, ICS, HDR, GcpE, DXPS, GPPS and MK (Supplementary Table 9). Moreover, the transcriptome expression analysis of these candidate transcripts showed that F01_transcript_23273, F01_transcript_22424, F01_transcript_52041, F01_transcript_21165, F01_transcript_8333, F01_transcript_19382, F01_transcript_7803, and F01_transcript_8589 were all highly expressed in the leaf tissue of *A. argyi* when compared to the other two tissues. Additionally, we analyzed the expression levels of DETs encoding enzymes in various pathways of terpenoid synthesis (Figure 3A). Certain genes, especially the F01_transcript_21165 and F01_transcript_8333, were more active in the leaves than roots and stems, suggesting they may be closely linked to terpenoid formation. Characterization of these genes should improve our understanding of the molecular mechanisms underlying the biosynthesis of terpenoids. These genes may be especially helpful for analyzing terpenoid metabolites in *A. argyi*. Finally, to evaluate the relative expression levels of the key putative genes involved in terpenoid biosynthesis, 12 candidate genes encoding vital enzymes, including TPS, FPS, GGPS, ICS and so on, were selected for qRT-PCR (Figure 3; Supplementary Table 9). The qRT-PCR results indicated the accuracy of transcriptome sequencing (Figure 3B).

Apart from structural genes, we also analyzed the regulatory genes, TFs and lncRNAs. Previous studies indicated that TFs can assist in augmenting the content of terpenoids and flavonoids by regulating the expression of multiple candidate genes related to their biosynthesis processes, and so TFs have drawn more attention in recent years. For example, the expression levels of Phenylalanine ammonia lyase (*PAL1*), (flavonol synthase) *FLS* and chalcone synthase (*CHS*) in *GmMYB12B2*-overexpressing plants were significantly higher than those in wild type *Arabidopsis* (Li et al., 2013). By activating promoters of the *CHS8* gene, the transcript factor *GmMYB29* advanced the biosynthesis of isoflavone (Chu et al., 2017), while *GmMYB39* inhibited isoflavone biosynthesis by repressing the transcription levels of CHS in soybean (Li et al., 2017). In this study, 5,604 candidate TFs were identified and assigned into 20 different TF families (Figure 4A), and we anticipate that these RLK-Pelle_CrRLK-1, RLK-Pelle_LRR-l-1 and RLK-Pelle_DLSV families of TFs somehow contribute to regulating terpenoid biosynthesis. Using genetic engineering methods to control TFs has proven potential value and has wide application prospects when investigating the regulation of terpenoid biosynthesis in *A. argyi*. Therefore, our findings may help improve future studies aiming to increase the yield of terpenoids via gene regulation and the production of transgenic plants.

## Conclusion

This study is the first to fully explore the transcriptome analysis of the *A. argyi* plant and to generate high-quality RNA-Seq data from its root, stem, and leaf tissues. In all, 36,820 non-redundant transcripts were identified. Among them, both candidate genes and TFs encoding key enzymes might be involved in the terpenoid biosynthesis pathway. These results can inform future research into the molecular mechanisms involved in terpenoid synthesis and how to increase terpenoids' yield in *A. argyi* leaves via gene regulation and genetic engineering.

## Data Availability Statement

The data presented in the study are deposited in the NCBI BioProject database, accession numbers PRJNA722755 and PRJNA722539. The final assembly and FPKM data were submitted to Dryad with doi: 10.5061/dryad.573n5tb7j.

## Author Contributions

YC and KZ designed the experiments. XG, JW, and ZS carried out the experiments. YC, ZZho, and ZZha analyzed the experimental results. YC wrote the manuscript. All authors have read, edited, and approved the current version of the manuscript.

## Conflict of Interest

The authors declare that the research was conducted in the absence of any commercial or financial relationships that could be construed as a potential conflict of interest. The reviewer YY declared a shared affiliation, with no collaboration, with one of the author ZZha to the handling editor at the time of the review.

## Supplementary Material

Supplementary Figure 1 Flowchart of bioinformatics analysis of full-length transcriptome.

Supplementary Figure 2 Busco results.

Supplementary Figure 3 The length distribution of Reads of Insert (ROI) from the SMRT data.

Supplementary Table 1 Primers for qRT-PCR and the amplification efficiency of the primers in this study.

Supplementary Table 2 Overview of assembly and quality evaluation of the *A. argyi* RNA-seq clean data.

Supplementary Table 3 Functional annotation of *A. argyi* transcripts against eight different public databases.

Supplementary Table 4 Go annotation of *A. argyi* transcripts.

Supplementary Table 5 Transcripts expressed in the root, stem and leaf tissues of *A. argyi*.

Supplementary Table 6 Transcripts specific-expressed in the *A. argyi* leaf tissue.

Supplementary Table 7 Expression profiles of 13850 DETs between leaf and root of *A. argyi*.

Supplementary Table 8 Expression profiles of 9502 DETs between leaf and stem of *A. argyi*.

Supplementary Table 9 One hundred and eighty-seven genes involved in terpenoid biosynthesis in *A. argyi*.

Supplementary Table 10 Thirty-two candidate genes involved in terpenoid biosynthesis.

Supplementary Table 11 Predicted lncRNA of *A. argyi*.

Supplementary Table 12 Candidates AS.
