# Supplementary material for: Full-Length Transcriptome Analysis Reveals Candidate Genes Involved in Terpenoid Biosynthesis in Artemisia argyi
Source: Front Genet. 2021 Jun 22;12:659962. doi: 10.3389/fgene.2021.659962 (PMC8258318; doi:10.3389/fgene.2021.659962)
Supplement: Supplementary Figure 1 — Flowchart of bioinformatics analysis of full-length transcriptome. [file Data_Sheet_1.ZIP › Table S3 Function annotation of A. argyi transcripts against eight different public databases..docx]

**Table S3. Annotation of transcripts against eight different public databases.**

| Annotated database | Number of annotated transcripts | Annotated transcripts ratio (%) | 300<=length<1000 | length>=1000 |
| --- | --- | --- | --- | --- |
| COG | 16,993 | 46.15 | 692 | 16,299 |
| GO | 22,965 | 62.37 | 1,528 | 21,424 |
| KEGG | 15,688 | 42.61 | 1,033 | 14,643 |
| KOG | 22,404 | 60.85 | 1,187 | 21,204 |
| Pfam | 29,721 | 80.75 | 1,680 | 28,038 |
| SwissProt | 27,548 | 74.82 | 1,577 | 25,954 |
| eggNOG | 33,807 | 91.82 | 2,059 | 31,731 |
| Nr | 34,599 | 93.97 | 2,217 | 32,359 |
| All | 34,839 | 94.62 | 2,235 | 32,578 |
