# Supplementary material for: Full-Length Transcriptome Analysis Reveals Candidate Genes Involved in Terpenoid Biosynthesis in Artemisia argyi
Source: Front Genet. 2021 Jun 22;12:659962. doi: 10.3389/fgene.2021.659962 (PMC8258318; doi:10.3389/fgene.2021.659962)
Supplement: Supplementary Figure 1 — Flowchart of bioinformatics analysis of full-length transcriptome. [file Data_Sheet_1.ZIP › Table S1 Primers for qRT-PCR and the amplification efficiency of the primers in this study.docx]

**Table S1. Primers for qRT-PCR and the amplification efficiency of the primers in this study.**

| **Primer** | **Forward primer (5'-3')** | **Reverse primer (5'-3')** | **amplification efficiency (%)** |
| --- | --- | --- | --- |
| *AaActin* | GCCATACTAGGCGTACTCGA | TTGGTTGGGCTTGTAAATAG | 95.2 |
| *F01_transcript_33110* | AGAGTATACTAGGGCAGTGTT | GACACCCTTTGACACCATGT | 90.1 |
| *F01_transcript_14972* | TTGATAAATCTATATGAAGC | CAGTGCATGATTTATCAGTTC | 92.5 |
| *F01_transcript_9751* | TGTTAAAGTGGCGGATGCATTG | GATGAGTCCTGTGTCTCAA | 99.3 |
| *F01_transcript_16752* | TCTGCTATATTTCAGTGTATA | AGGGTGAATAACTATATCAATA | 98.3 |
| *F01_transcript_10409* | TGAATCGGGGATTATCTGTTG | GTCCATGATATCATCAAGCACA | 95.4 |
| *F01_transcript_16157* | TGGCTGCAGCTCATATTTCT | CAGATTGTATCGACTCCCA | 99.5 |
| *F01_transcript_50745* | TTCAACCTGCCAACCTGGT | ATTATTCCAGTTGGCCCTCTCT | 98.9 |
| *F01_transcript_13259* | TGAGATGCCTGTTGGTTATGT | GAGCTCGAGTCATTCCATCT | 101.2 |
| *F01_transcript_4164* | TAAGGACGTGGCTGCAACTG | TGTTGATAGTCATCATCTGT | 103.4 |
| *F01_transcript_4816* | CGCAACGGTGTCCCACTCCAT | ATCTTCGATATCCCATGAATC | 100.0 |
| *F01_transcript_50072* | TGTTTTGAAATCAAGAACA | AATATGCTCAAGTTCCTGTTGAT | 96.4 |
| *F01_transcript_37391* | AGTGGTGAATGGTCAAGGAG | CATTTTGTCGGCTGCAATTTCA | 98.4 |
