# Supplementary material for: Full-Length Transcriptome Analysis Reveals Candidate Genes Involved in Terpenoid Biosynthesis in Artemisia argyi
Source: Front Genet. 2021 Jun 22;12:659962. doi: 10.3389/fgene.2021.659962 (PMC8258318; doi:10.3389/fgene.2021.659962)
Supplement: Supplementary Figure 1 — Flowchart of bioinformatics analysis of full-length transcriptome. [file Data_Sheet_1.ZIP › Table S2 Overview of assembly and quality evaluation of the A. argyi RNA-seq clean data..docx]

**Table S2. Overview of assembly and quality evaluation of the *A. argyi* RNA-seq clean data.**

| Sample ID | Obtained Reads | Obtained Base (bp) | GC(%) | Q20(%) | Q30(%) |
| --- | --- | --- | --- | --- | --- |
| Leaf1 | 19,167,575 | 5,734,529,702 | 43.00 | 98.06 | 94.38 |
| Leaf2 | 19,941,674 | 5,965,749,592 | 43.01 | 98.03 | 94.35 |
| Leaf3 | 19,394,783 | 5,804,348,548 | 42.92 | 97.78 | 93.68 |
| Stem1 | 19,223,322 | 5,745,884,166 | 42.76 | 97.89 | 93.95 |
| Stem2 | 21,217,782 | 6,340,234,082 | 42.84 | 98.06 | 94.39 |
| Stem3 | 19,576,569 | 5,846,163,348 | 42.83 | 97.89 | 93.96 |
| Root1 | 19,107,272 | 5,717,228,048 | 42.49 | 98.17 | 94.56 |
| Root2 | 25,479,768 | 7,619,899,580 | 42.46 | 97.89 | 93.94 |
| Root3 | 22,174,229 | 6,629,901,930 | 42.70 | 97.95 | 94.13 |
