# Supplementary figures and images for: Full-Length Transcriptome Analysis Reveals Candidate Genes Involved in Terpenoid Biosynthesis in Artemisia argyi
Source: Front Genet. 2021 Jun 22;12:659962. doi: 10.3389/fgene.2021.659962 (PMC8258318; doi:10.3389/fgene.2021.659962)

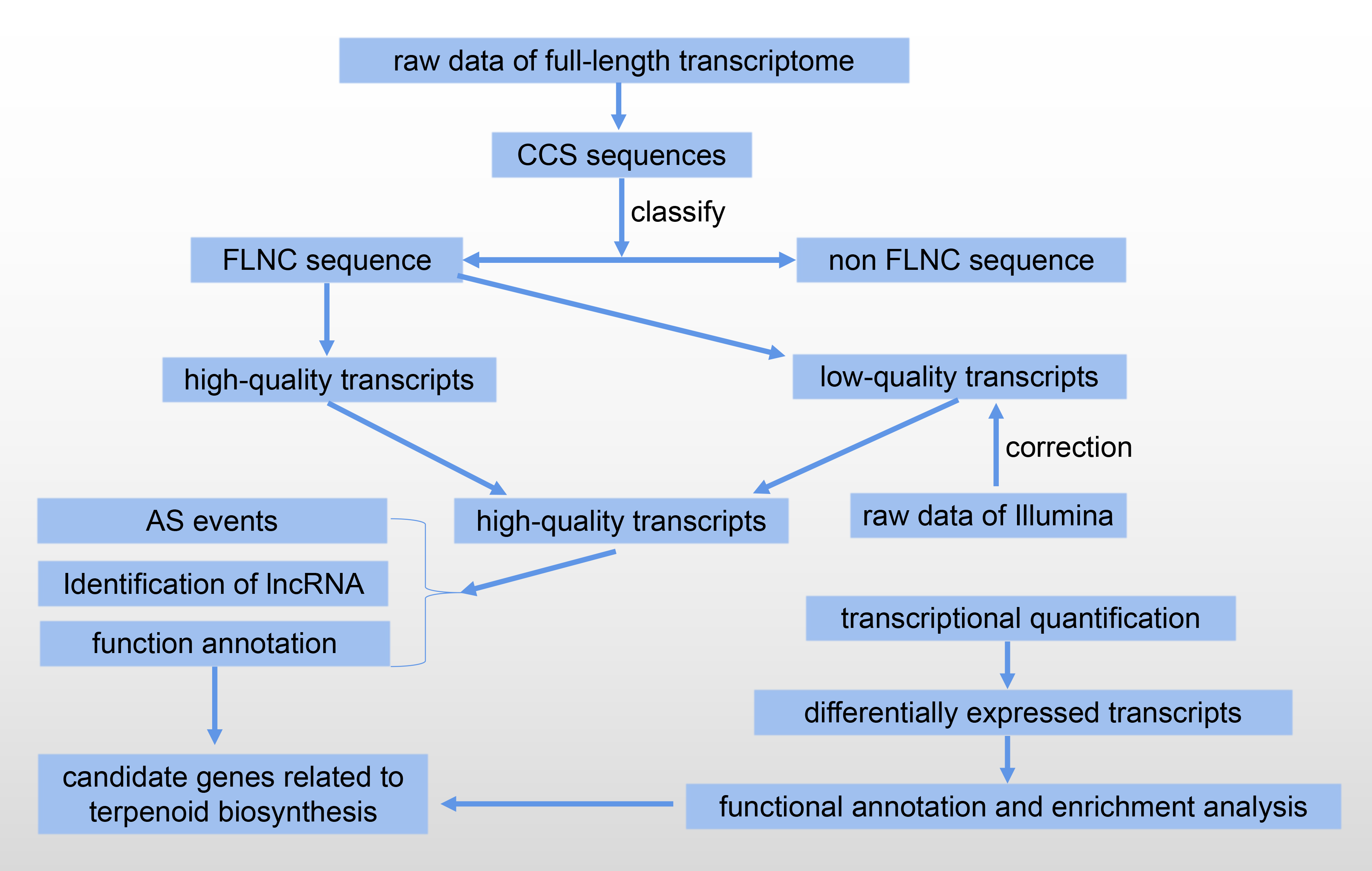

Supplement: Supplementary Figure 1 — Flowchart of bioinformatics analysis of full-length transcriptome. [file Data_Sheet_1.ZIP › Figure S1 Flowchart of bioinformatics analysis of full-length transcriptome.tif]

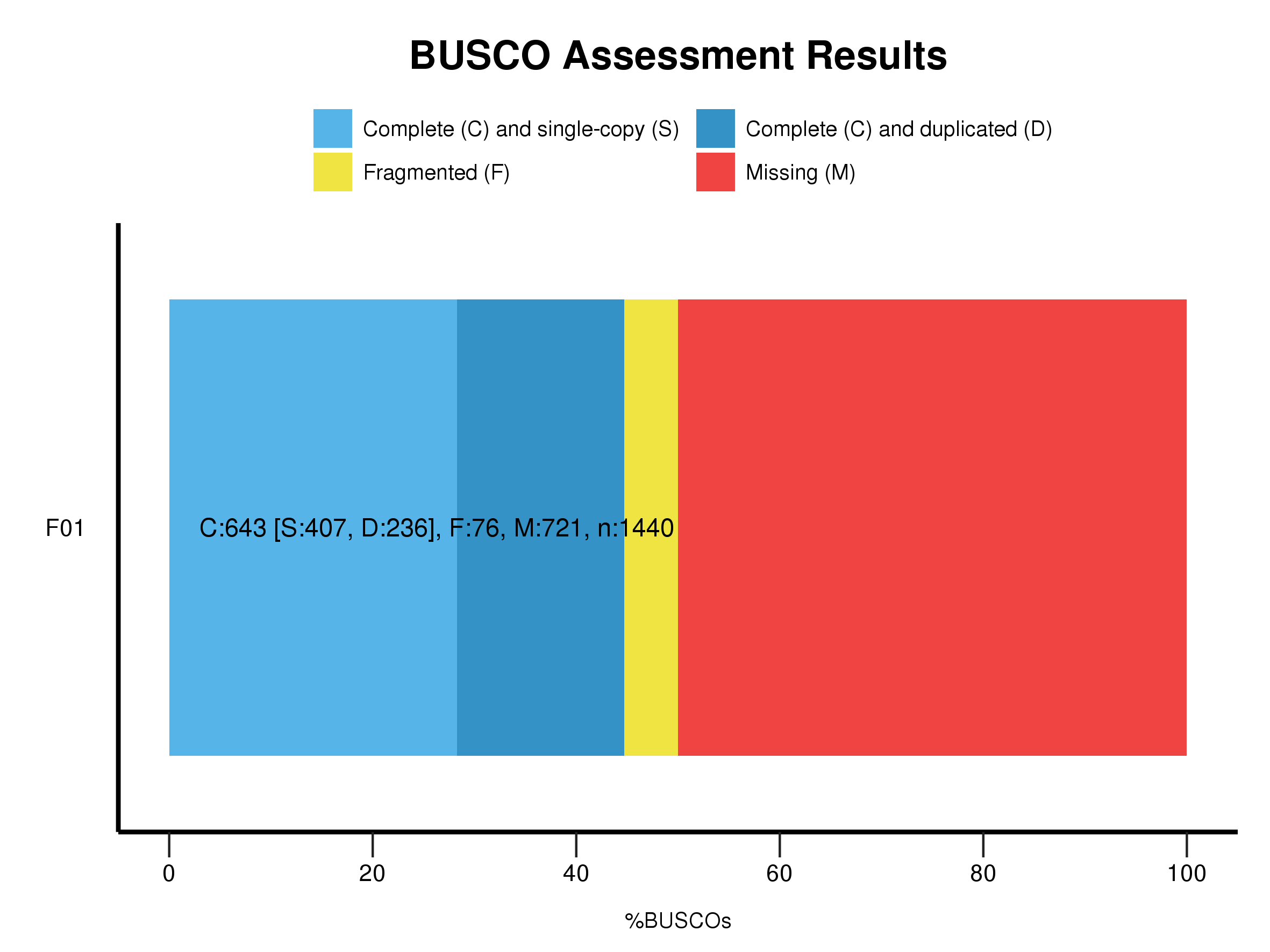

Supplement: Supplementary Figure 1 — Flowchart of bioinformatics analysis of full-length transcriptome. [file Data_Sheet_1.ZIP › Figure S2 busco.png]

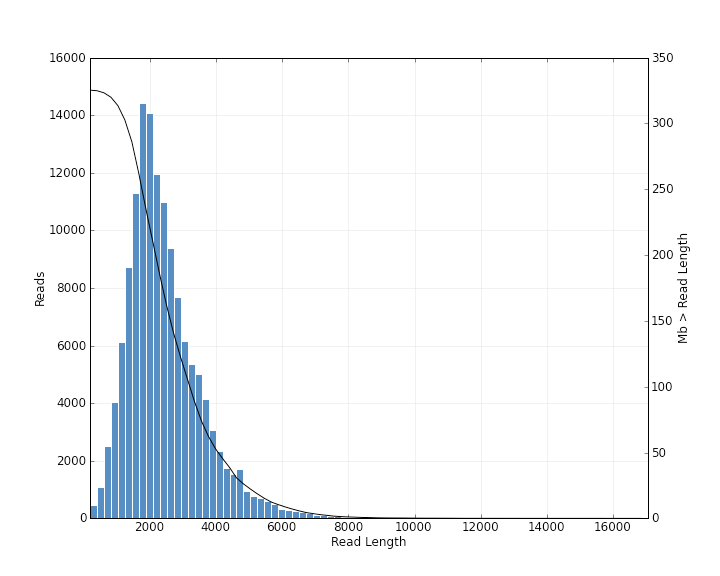

Supplement: Supplementary Figure 1 — Flowchart of bioinformatics analysis of full-length transcriptome. [file Data_Sheet_1.ZIP › Figure S3 Hist of ROI read length..tif]
